# Therapeutic Plasma Exchange in COVID-19-Associated Sepsis: IL-6 Dynamics, Inflammatory Phenotypes, and Short-Term Organ-Failure Trajectories in a Real-World Cohort

**DOI:** 10.3390/jcm15010010

**Published:** 2025-12-19

**Authors:** Nicoleta Sgavardea, Dorel Sandesc, Tamara Mirela Porosnicu, Ovidiu Bedreag, Ciprian Gîndac, Marius Papurica, Elena Hogea, Patricia Hogea, Iulia Georgiana Bogdan, Voichita Elena Lazureanu

**Affiliations:** 1 Doctoral School, “Victor Babes” University of Medicine and Pharmacy, Eftimie Murgu Square 2, 300041 Timisoara, Romania; nicoleta.cotaia@umft.ro; 2 Anaesthesia and Intensive Care Research Center, Faculty of Medicine, “Victor Babes” University of Medicine and Pharmacy, Eftimie Murgu Square 2, 300041 Timisoara, Romania; sandesc.dorel@umft.ro (D.S.); ciprian.gindac@umft.ro (C.G.); marius.papurica@umft.ro (M.P.); 3 Discipline of Microbiology, “Victor Babes” University of Medicine and Pharmacy, Eftimie Murgu Square 2, 300041 Timisoara, Romania; hogea.elena@umft.ro (E.H.); iulia-georgiana.bogdan@umft.ro (I.G.B.); lazureanu.voichita@umft.ro (V.E.L.); 4 Center for Research and Innovation in Precision Medicine of Respiratory Diseases (CRIPMRD), “Victor Babes” University of Medicine and Pharmacy, Eftimie Murgu Square 2, 300041 Timisoara, Romania; hogea.patricia@umft.ro

**Keywords:** COVID-19, sepsis, plasma exchange, interleukin-6, biomarkers

## Abstract

**Background and Objectives:** In severe COVID-19-associated sepsis, therapeutic plasma exchange (TPE) is used as a rescue strategy to modulate cytokine and coagulation derangements, but its biomarker and organ-failure effects remain incompletely characterised. We evaluated peri-procedural changes in interleukin-6 (IL-6), other inflammatory markers, and Sequential Organ Failure Assessment (SOFA) scores according to TPE intensity, timing, and inflammatory phenotypes. **Methods:** We conducted a single-centre retrospective cohort study including 102 mechanically ventilated adults with COVID-19-associated sepsis who received ≥1 TPE session. Patients were grouped by number of sessions (1, 2, ≥3), timing (≤14 vs. >14 days from symptom onset), IL-6 responder status (≥50% reduction), and two unsupervised inflammatory–thrombotic clusters. Peri-procedural changes (Δ) in biomarkers and SOFA were compared using non-parametric tests, with multivariable logistic and linear regression exploring predictors of IL-6 response and ΔSOFA. **Results:** Baseline severity was similar across TPE-intensity groups, with median APACHE II scores of 11–12 and SOFA scores around 7 in all strata. Median IL-6 concentrations declined after TPE in each group (e.g., Δ −59.4 pg/mL after 1 session and Δ −65.1 pg/mL after ≥3 sessions), but between-group differences in ΔIL-6 were not statistically significant (*p* = 0.276). By contrast, D-dimer exhibited a marked decline only in the 1-session group (median Δ −1.7 mg/L vs. ~0.0 mg/L in the 2- and ≥3-session groups; *p* < 0.001). Timing (early vs. late TPE) did not materially affect ΔIL-6, ΔCRP, ΔSOFA (median 0.0 in both), or ΔD-dimer. Overall, 50% of patients were IL-6 responders; baseline IL-6 was the only independent predictor (adjusted OR 1.9 per doubling, 95% CI 1.3–2.8). A hyperinflammatory–thrombotic cluster (*n* = 44) exhibited higher baseline IL-6 (612.3 vs. 92.4 pg/mL), more ≥3-session TPE (65.9% vs. 29.3%), and higher IL-6 responder rates (75.0% vs. 31.0%), but similar 28-day mortality (40.9% vs. 29.3%). **Conclusions:** In this real-world TPE programme, biochemical improvements—particularly IL-6 and D-dimer reductions in hyperinflammatory–thrombotic patients—were not consistently accompanied by short-term SOFA or survival benefits, underscoring the need for phenotype-guided and trial-based use.

## 1. Introduction

Sepsis and severe coronavirus disease 2019 (COVID-19) converge on a common endpoint of dysregulated host response, microcirculatory failure, and progressive multi-organ dysfunction as captured in contemporary Sepsis-3 definitions of viral and bacterial sepsis [1]. In SARS-CoV-2 infection, endothelial activation, diffuse microvascular thrombosis, and complement-mediated injury drive a characteristic “immunothrombotic” phenotype that links pulmonary and systemic organ failure [2]. At the same time, a minority of patients progress to a hyperinflammatory “cytokine storm” state with striking elevations of interleukin-6 (IL-6), C-reactive protein (CRP), ferritin, and D-dimer, which strongly correlate with respiratory failure and death [3]. Although standard sepsis bundles focus on antimicrobial therapy, source control, and organ support, these pathophysiological insights have renewed interest in adjunctive strategies that directly modulate the circulating inflammatory and coagulopathic milieu.

Among circulating mediators, IL-6 has emerged as one of the most reproducible biomarkers of COVID-19 severity. A meta-analysis of more than 1400 patients found that IL-6 concentrations were approximately threefold higher in complicated versus uncomplicated COVID-19 and were associated with increased risk of ICU admission and mortality [4]. Prospective cohorts have subsequently confirmed that early IL-6 levels predict progression to acute respiratory distress syndrome (ARDS), need for mechanical ventilation, and prolonged hospitalisation, supporting its use for risk stratification and dynamic monitoring [5]. In parallel, randomised trials and meta-analyses of IL-6 receptor blockade (tocilizumab, sarilumab) have shown modest but clinically meaningful reductions in 28-day mortality when these agents are added to corticosteroids in hypoxic, systemically inflamed patients, underscoring the central role of IL-6–driven immunopathology in severe disease [6]. However, pharmacologic IL-6 inhibition is not universally effective, may be limited by access, and is typically delivered as a one-off or short course intervention rather than as a titratable, circuit-based therapy.

Therapeutic plasma exchange (TPE) is biologically attractive in this context. By removing patient plasma and replacing it with albumin and/or fresh frozen plasma, TPE can simultaneously clear pro-inflammatory cytokines, damage-associated molecular patterns, autoantibodies, and pro-coagulant proteins, while replenishing anticoagulant, anti-complement, and endothelial-protective factors [3,7]. TPE is already an established treatment for other cytokine-storm conditions, such as thrombotic microangiopathies and haemophagocytic lymphohistiocytosis, in which it can rapidly debulk circulating mediators and stabilise organ function. Extrapolating from this experience, several expert groups have proposed TPE as one of several extracorporeal blood purification (EBP) options for COVID-19-associated hyperinflammation, alongside haemoadsorption, high cut-off membranes, and adsorption-capable dialysis filters [7,8,9,10]. Small COVID-19 series and case reports have described rapid falls in IL-6, CRP, and D-dimer after TPE, occasionally accompanied by transient haemodynamic and oxygenation improvement, but these observations are heterogeneous and often uncontrolled [3,11].

In addition to IL-6, several other biomarkers are routinely monitored in severe COVID-19 and viral sepsis. CRP and ferritin reflect hepatic acute-phase responses and macrophage activation, while fibrinogen, D-dimer, and lactate dehydrogenase capture the intertwined coagulopathic and tissue-injury components of the “immunothrombotic” phenotype [2,3]. Marked elevations and dynamic changes in these markers have been associated with progression to ARDS, venous thromboembolism, and death, and they are therefore frequently used to guide escalation of anti-inflammatory and anticoagulant therapies. For these reasons, we evaluated peri-procedural changes in CRP, ferritin, D-dimer, and other acute-phase reactants alongside IL-6 in our TPE programme [11,12].

Beyond TPE, a broader EBP literature has developed rapidly during the pandemic. Reviews and expert recommendations highlight that TPE devices can attenuate circulating cytokines and endotoxin, with reported reductions in IL-6 and CRP and signals toward improved vasopressor requirements in selected patients [7,8,9,13,14,15]. Nevertheless, most studies are small, single-centre, and at high risk of bias, and randomised data are scarce. Existing guidance therefore frames EBP as a rescue or adjunctive therapy for carefully selected patients with refractory shock and hyperinflammation rather than as a standard component of sepsis care [7,8,9,10,14,15]. A recurring message across this literature is that patient selection, timing, and intensity of treatment may be at least as important as the specific device chosen.

Within this expanding EBP landscape, TPE occupies a distinctive niche because it combines non-selective cytokine clearance with replacement of physiologic plasma constituents. Observational cohorts of TPE in severe COVID-19 have reported substantial reductions in IL-6, CRP, erythrocyte sedimentation rate, and other acute-phase reactants after one or more sessions, but often with persistently high mortality, raising the possibility that biochemical improvements do not consistently translate into survival benefit [11]. In a single-centre cohort of 65 ICU patients with severe SARS-CoV-2 infection, Porosnicu et al. showed that one to >2 TPE sessions led to marked IL-6 declines (e.g., from ~300 to ~150 pg/mL in patients receiving >2 sessions) and improved ROX indices, yet 72% of patients died and survival did not differ by number of sessions [11]. A randomised controlled trial of TPE as an adjunct to standard care in life-threatening COVID-19 suggested improvements in organ-failure scores and inflammatory markers compared with standard care alone but was underpowered for definitive mortality conclusions and applied an empirical course of up to five sessions without biomarker-guided tailoring [12,14,15].

Several key questions therefore remain unresolved. First, the optimal “dose” of TPE—expressed as the number of sessions, interval between procedures, and plasma volume exchanged—has not been standardised. Most centres perform empirical courses of three to five sessions, extrapolating from thrombotic microangiopathies and other apheresis indications rather than from sepsis-specific pharmacodynamic data [7,8,9,11,12]. Second, the timing of TPE within the disease trajectory may be critical. COVID-19-associated sepsis evolves from early viral replication and hyperinflammation to secondary infections and immunoparesis; whether early or late TPE yields greater benefit, or whether specific inflammatory “windows” exist in which TPE is most effective, is unknown [2,3,7]. Third, not all patients exhibit the same biochemical response to TPE. Some experience steep cytokine declines, whereas others show modest or paradoxical changes. Identifying clinical or laboratory features that define “responders”—for example, baseline IL-6 levels, D-dimer, or organ-failure scores—could help refine indications and avoid unnecessary procedures [4,5,11,12].

In particular, IL-6 has emerged as a candidate biomarker because of its central role in COVID-19 pathophysiology, its strong association with disease severity, and its use for risk stratification and eligibility for IL-6–targeted drugs [4,5,6]. However, whether TPE-induced reductions in IL-6 mirror the effects of pharmacologic IL-6 inhibition on downstream organ function—or instead simply mark nonspecific dilution or clearance—remains uncertain. Finally, there is limited information on the coupling between biomarker trajectories and short-term organ-failure dynamics in real-world TPE programmes. A therapy may normalise laboratory parameters without translating into meaningful Sequential Organ Failure Assessment (SOFA) improvement, raising questions about its true clinical impact [7,8,9,11]. By analysing real-world data from a single coordinated programme of TPE in COVID-19-associated sepsis, we sought to explore these knowledge gaps in a hypothesis-generating fashion.

The primary objective of this study was to describe how IL-6 and other inflammatory markers evolve before and after TPE and to assess whether the number of TPE sessions modifies these trajectories. Secondary objectives were to evaluate the association of treatment timing with biomarker and SOFA changes and to identify baseline predictors of a strong IL-6 response. We hypothesised that (i) TPE would reduce IL-6 and D-dimer levels, (ii) more sessions and earlier initiation would augment this effect, and (iii) IL-6 reduction would correlate with early organ-failure improvement, thereby informing future biomarker-guided TPE strategies in COVID-19-associated sepsis.

## 2. Materials and Methods

### 2.1. Study Design & Setting

We conducted a prospective study observational cohort study in adult patients with severe COVID-19-associated sepsis who underwent TPE as part of routine clinical care between April 2020 and April 2024 in the adult intensive care units of a single tertiary university hospital affiliated with the Victor Babes University of Medicine and Pharmacy. TPE was adopted as an adjunctive rescue strategy for selected patients with refractory inflammation or organ dysfunction. The present report adheres to the STROBE (Strengthening the Reporting of Observational Studies in Epidemiology) statement for cohort studies [16]. The study population was derived from the adult medical–surgical intensive care units of a single tertiary university hospital, which operated under shared institutional protocols for COVID-19 management and adjunctive extracorporeal therapies. Within this network of ICUs, TPE was adopted as an adjunctive rescue strategy for selected patients with refractory inflammation or organ dysfunction.

All consecutive patients receiving at least one TPE session for COVID-19-associated sepsis were eligible for inclusion. Data were extracted from electronic medical records and prospectively maintained local registries. The analysis focused on peri-procedural dynamics, capturing clinical and laboratory parameters immediately before the first TPE session (“baseline”) and immediately after the last session in the planned course (“post-TPE”). Because this was a secondary analysis of anonymised data, patient management was unaffected by the study.

A total of 935 adults with confirmed SARS-CoV-2 infection and suspected sepsis or septic shock were screened during the study period. Of these, 116 received at least one TPE session. After excluding patients with missing core laboratory data (baseline or post-TPE IL-6 or CRP) or incomplete documentation of TPE exposure, 102 patients remained in the final analysis cohort.

### 2.2. Patient Population and TPE Protocol

Adult patients (≥18 years) with microbiologically confirmed SARS-CoV-2 infection and sepsis or septic shock (defined by organ dysfunction and/or vasopressor requirement) who received ≥1 TPE session were included. All patients were mechanically ventilated or received high-flow oxygen at the time of TPE. Exclusion criteria for the present analysis were missing core laboratory data (IL-6 or CRP) at either baseline or post-TPE, or incomplete documentation of TPE exposure.

TPE was typically initiated for one or more of the following indications: very high or rising IL-6 and D-dimer levels despite guideline-directed therapy; refractory vasopressor requirement; or rapidly worsening respiratory or multi-organ failure interpreted as hyperinflammatory. TPE sessions were performed using standard centrifugal or membrane-based apheresis devices available in our institution, targeting an exchange of approximately 1.0–1.5 estimated plasma volumes per procedure. Replacement fluid consisted predominantly of 5% albumin, supplemented with fresh frozen plasma in patients with coagulopathy or at increased bleeding risk, according to clinician judgement. Sessions typically lasted 2–3 h and were performed with regional citrate or systemic heparin anticoagulation as per local practice. The decision to perform 1, 2, or ≥3 sessions, as well as spacing between sessions, was left to the treating intensivist, reflecting real-world practice.

All patients received guideline-directed COVID-19 management according to contemporaneous institutional protocols, including systemic corticosteroids (typically dexamethasone 6–8 mg/day or an equivalent glucocorticoid regimen), prophylactic or therapeutic-dose low-molecular-weight heparin, and empirical broad-spectrum antibiotics when bacterial coinfection was suspected. Use of IL-6 receptor antagonists (tocilizumab or sarilumab), remdesivir, and other immunomodulators was at the discretion of the treating team and constrained by national drug availability. Exposure to these co-interventions was abstracted at baseline and summarised across the TPE-intensity groups.

These indications were formalised in a local consensus document that recommended considering TPE in patients with severe COVID-19-associated sepsis who exhibited marked inflammatory or thrombotic activation despite guideline-directed therapy. However, the ultimate decision to initiate TPE, as well as the number and spacing of sessions, remained at the discretion of the attending ICU physician.

### 2.3. Data Collection and Definitions

Demographics (age, sex), comorbidities (diabetes mellitus, arterial hypertension, obesity, chronic obstructive pulmonary disease), and time from symptom onset to first TPE were retrieved. Illness severity at baseline was assessed using APACHE II and SOFA scores. Respiratory failure was characterised by the PaO_2_/FiO_2_ ratio, while haemodynamic status was approximated by mean arterial pressure (MAP) and the need for norepinephrine. Vital signs and organ-support variables were recorded at baseline and after the final TPE session. In addition, we recorded key COVID-19-directed therapies administered before or at the time of the first TPE session, including systemic corticosteroid use and dose, anticoagulation intensity (prophylactic vs. therapeutic), administration of IL-6 receptor antagonists, remdesivir therapy, and empirical or targeted antibiotics. These variables were used to characterise the background standard of care in each TPE-intensity group.

Inflammatory and coagulation biomarkers assessed at both timepoints included IL-6, CRP, ferritin, D-dimer, white blood cell count, lymphocyte percentage, fibrinogen, lactate dehydrogenase, and procalcitonin. Lactate values, when available, were also extracted. For each marker, we calculated the absolute change (post-TPE minus baseline). A priori, a “strong IL-6 response” was defined as a ≥50% reduction from baseline to post-TPE, allowing stratification into responders and non-responders. Timing of TPE was characterised by the number of days from symptom onset to the first TPE session and dichotomised into early (≤14 days) and late (>14 days).

### 2.4. Statistical Analysis

Continuous variables were inspected for distributional features and are presented as median and interquartile range (IQR). Categorical variables are reported as counts and percentages. Patients were categorised into three exposure groups according to the number of TPE sessions received: 1, 2, or ≥3. Baseline continuous characteristics and biomarker values were compared across these groups using the Kruskal–Wallis test; categorical variables were compared using χ^2^ tests without continuity correction. For key pre–post comparisons within groups, we primarily interpreted changes descriptively, whereas between-group differences in change (Δ values) were formally tested with Kruskal–Wallis.

To explore timing effects, early versus late TPE groups were compared using the Mann–Whitney test for continuous Δ variables and χ^2^ tests for categorical variables. The IL-6 responder versus non-responder comparison used the same approach. Associations between changes in IL-6 and changes in CRP, D-dimer, PaO_2_/FiO_2_, SOFA, and lactate were quantified using Spearman’s rank correlation. Two-sided *p*-values < 0.05 were considered statistically significant.

### 2.5. Inflammatory–Thrombotic Clustering

To explore inflammatory–thrombotic phenotypes, we performed an unsupervised clustering analysis using baseline IL-6, CRP, ferritin, and D-dimer concentrations. Continuous variables were log-transformed where appropriate to reduce skewness and then standardised to z-scores before clustering. We applied agglomerative hierarchical clustering with Ward’s minimum-variance linkage and Euclidean distance. Visual inspection of the dendrogram and the relative gain in within-cluster homogeneity supported a two-cluster solution, which was subsequently labelled post hoc as “hyperinflammatory–thrombotic” and “hypoinflammatory” based on their biomarker profiles. These clusters were then compared with respect to TPE exposure, IL-6 responder status, D-dimer changes, and 28-day mortality.

## 3. Results

Table 1 summarises baseline characteristics across patients receiving 1, 2, or ≥3 TPE sessions. Age and illness severity were broadly comparable, with median ages of 51.0, 53.5, and 49.0 years (*p* = 0.381) and similar APACHE II scores around 11–12 and SOFA scores around 7 in all groups (*p* = 0.080 and *p* = 0.454, respectively). Respiratory impairment was severe across the cohort, with baseline PaO_2_/FiO_2_ medians of 90.0, 110.0, and 115.0 mmHg (*p* = 0.263). The main difference between groups was timing of TPE: patients receiving ≥3 sessions started later after symptom onset (median 18.0 days) compared with 12.0 days for 1 session and 15.0 days for 2 sessions (*p* = 0.005). Baseline IL-6 showed a trend toward higher levels in the ≥3-session group (305.5 pg/mL, IQR 53.2–1306.3) than in the 1-session (143.1 pg/mL) or 2-session (37.0 pg/mL) groups (*p* = 0.056), while baseline CRP and comorbidities such as diabetes, hypertension, obesity, and COPD were similarly distributed (all *p* > 0.28). Background COVID-19-directed therapies, including systemic corticosteroids, therapeutic anticoagulation, IL-6 receptor blockade, remdesivir, and empirical antibiotics were broadly similar across TPE-intensity strata, with no obvious systematic imbalances.

Table 2 describes peri-procedural changes in inflammatory biomarkers according to the number of TPE sessions. IL-6 concentrations decreased in all groups, with median ΔIL-6 values of −59.4 pg/mL (IQR −447.6 to 0.6) after 1 session, −1.8 pg/mL (−31.2 to 0.9) after 2 sessions, and −65.1 pg/mL (−748.5 to 53.9) after ≥3 sessions, although differences in IL-6 change across groups were not statistically significant (*p* = 0.276). CRP and ferritin also showed modest median reductions of roughly −7 to −8 mg/L and −72.0 to −193.5 µg/L, respectively, without significant between-group differences (*p* = 0.767 and *p* = 0.162). By contrast, D-dimer exhibited a marked and statistically significant decline only in the 1-session group (median Δ −1.7 mg/L, IQR −3.0 to −0.3), while changes were minimal in the 2- and ≥3-session groups (Δ −0.1 and 0.0 mg/L; *p* < 0.001 for Δ across groups). White blood cell counts and lymphocyte percentages remained essentially stable around baseline values in all groups, with median Δ values close to 0 and *p*-values ≥ 0.865, indicating that TPE had little impact on overall leukocyte profiles.

Table 3 reports haemodynamic, respiratory, and lactate changes across TPE exposure groups. Median baseline MAP was similar at 79.0, 80.0, and 77.0 mmHg in the 1-, 2-, and ≥3-session groups, respectively, but post-TPE trends diverged: patients receiving ≥3 sessions showed a modest MAP increase (Δ +2.0 mmHg, IQR −5.0 to 9.0), whereas those receiving 1 or 2 sessions had small declines (Δ −1.0 and −2.0 mmHg). The between-group difference in MAP change was statistically significant (*p* = 0.045), suggesting a possible haemodynamic stabilisation with more intensive TPE. PaO_2_/FiO_2_ ratios improved slightly in all groups, with median Δ values of +10.0, +6.0, and +7.0 mmHg, but these changes were modest and not significantly different across groups (*p* = 0.701). Serum lactate decreased by a median of −0.1 mmol/L in every group, with overlapping IQRs and a non-significant *p*-value (0.713), indicating only minor global shifts in tissue perfusion markers over the TPE course.

Table 4 compares peri-procedural changes in IL-6, CRP, SOFA score, and D-dimer between patients receiving early (≤14 days from symptom onset) versus late (>14 days) TPE. Median IL-6 reductions were modest and similar in both groups (−20.0 pg/mL, IQR −180.5 to 1.7 for early vs. −31.0 pg/mL, −612.0 to 24.1 for late; *p* = 0.875). CRP decreased by a median of −8.0 mg/L (−95.0 to 6.0) with early TPE and −7.0 mg/L (−72.0 to 11.0) with late TPE (*p* = 0.361). SOFA scores showed no net improvement overall, with median ΔSOFA of 0.0 (−1.0 to 0.0) in both groups (*p* = 0.957). D-dimer declined slightly by −0.2 mg/L (−1.8 to 0.4) in the early group and −0.2 mg/L (−1.8 to 0.3) in the late group, again without any meaningful difference (*p* = 0.985). Overall, within this cohort, the timing of TPE initiation relative to symptom onset did not substantially modify short-term biomarker or SOFA trajectories.

Table 5 presents Spearman correlations between changes in IL-6 and concurrent changes in key clinical and laboratory variables. Across 102 patients, the association between ΔIL-6 and ΔSOFA was essentially null (ρ = 0.03, *p* = 0.778), indicating that reductions in IL-6 did not consistently track with short-term organ-failure improvement. Correlations with ΔCRP (ρ = 0.16, *p* = 0.113), ΔD-dimer (ρ = 0.00, *p* = 0.987), and ΔPaO_2_/FiO_2_ (ρ = 0.11, *p* = 0.267) were weak and non-significant, suggesting only loose coupling between IL-6 dynamics and other inflammatory or respiratory markers. In the subset with lactate data (*n* = 87), the correlation between ΔIL-6 and Δlactate was likewise negligible (ρ = −0.01, *p* = 0.92). Collectively, these results indicate that biochemical improvement in IL-6 alone is a poor standalone surrogate for early organ support or coagulation changes in this real-world TPE programme. These correlation analyses were further limited by incomplete availability of some variables, particularly lactate, which was missing in a subset of patients and may have attenuated detectable associations.

Table 6 compares baseline characteristics between IL-6 responders (≥50% reduction) and non-responders. Age distributions were similar, with median values of 48.5 versus 54.0 years (*p* = 0.726), and timing of TPE did not differ substantially (median 15.0 vs. 13.0 days from symptom onset; *p* = 0.459). Baseline illness severity was broadly comparable, with APACHE II scores around 11–12 (*p* = 0.222) and SOFA scores of 8.0 (IQR 5.5–11.5) in responders versus 6.0 (5.0–10.0) in non-responders (*p* = 0.136). Baseline CRP levels were also overlapping at 92.6 mg/L (29.7–208.5) versus 81.0 mg/L (17.0–169.0; *p* = 0.286). The only parameter that clearly distinguished the two groups was baseline IL-6: responders had markedly higher starting levels (median 445.8 pg/mL, IQR 110.5–2081.5) than non-responders (50.0 pg/mL, 17.9–181.0; *p* < 0.001), supporting a strong relationship between initial cytokine burden and subsequent IL-6 clearance with TPE.

Table 7 shows the multivariable logistic regression model for IL-6 responder status (≥50% reduction). After simultaneous adjustment for all listed covariates in 102 patients, log_2_ baseline IL-6 was the only independent predictor of response: each doubling of baseline IL-6 was associated with an almost twofold higher odds of achieving a ≥50% reduction (adjusted OR 1.9, 95% CI 1.3–2.8; *p* = 0.002). Days from symptom onset to first TPE (OR 1.0, 95% CI 0.9–1.1; *p* = 0.614), baseline APACHE II score (OR 1.1, 0.9–1.2; *p* = 0.186), and baseline D-dimer (OR 1.1 per 1 mg/L, 0.9–1.3; *p* = 0.219) were not significantly associated with responder status. Likewise, the number of TPE sessions (2 vs. 1: OR 0.8; ≥3 vs. 1: OR 1.2) and early versus late TPE (OR 1.1, 0.5–2.4; *p* = 0.812) did not materially influence the probability of IL-6 response once baseline cytokine levels were taken into account.

Table 8 presents a multivariable linear regression model for ΔSOFA (post-TPE minus baseline), where negative values indicate organ-failure improvement. In this cohort of 102 patients, the intercept was close to zero (β −0.1, 95% CI −0.9 to 0.7; *p* = 0.786), and most predictors were not significantly associated with ΔSOFA. Baseline log_2_ IL-6 (β −0.1 per doubling, −0.3 to 0.1; *p* = 0.293), the magnitude of IL-6 decrease (β −0.1 per −100 pg/mL, −0.3 to 0.0; *p* = 0.071), days from symptom onset, the number of TPE sessions, and baseline D-dimer all showed small, non-significant coefficients. The only statistically significant predictor was baseline SOFA, where each additional point was associated with a modestly more favourable subsequent change (β −0.1, 95% CI −0.2 to 0.0; *p* = 0.044), consistent with slightly greater scope for improvement in patients starting with higher organ-failure scores. Overall, the model suggests that neither IL-6 levels nor TPE intensity strongly dictated short-term SOFA trajectories.

Table 9 contrasts two inflammatory–thrombotic phenotypes derived from unsupervised clustering. Cluster 1, labelled hyperinflammatory–thrombotic (*n* = 44), displayed substantially higher baseline IL-6 (median 612.3 pg/mL, IQR 255.6–1843.7) compared with Cluster 2 (hypoinflammatory, *n* = 58; 92.4 pg/mL, 28.1–204.8; *p* < 0.001). This cluster also had higher CRP (138.7 vs. 61.5 mg/L; *p* < 0.001), ferritin (1658.4 vs. 921.6 µg/L; *p* = 0.002), and D-dimer (3.8 vs. 1.3 mg/L; *p* < 0.001), confirming a more intense inflammatory and thrombotic milieu. Patients in Cluster 1 more frequently received ≥3 TPE sessions (65.9% vs. 29.3%; *p* = 0.001) and were much more likely to achieve IL-6 responder status (75.0% vs. 31.0%; *p* < 0.001). D-dimer decreased more in Cluster 1 (median Δ −0.8 mg/L, −2.5 to 0.1) than in Cluster 2 (−0.1 mg/L, −0.6 to 0.3; *p* = 0.014). Despite these biochemical advantages, 28-day mortality remained high and did not differ significantly between phenotypes (40.9% vs. 29.3%; *p* = 0.231), suggesting that more aggressive TPE in the hyperinflammatory–thrombotic cluster translated primarily into biomarker rather than survival differences.

**Figure 1:**
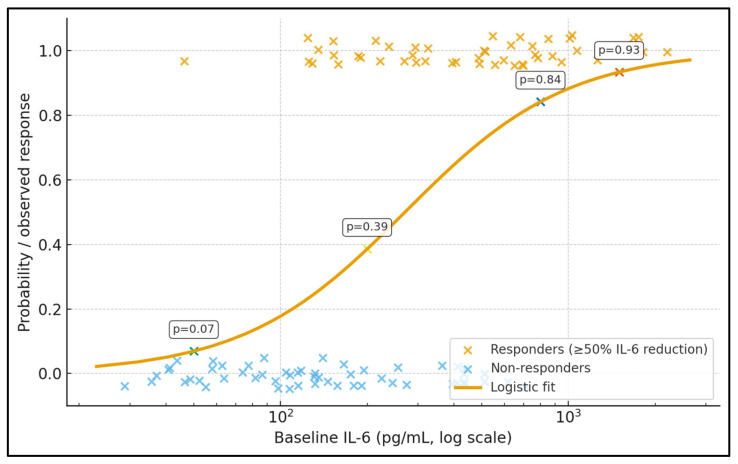
Baseline IL-6 and probability of IL-6 response to TPE.

Figure 1 illustrates the relationship between baseline IL-6 (log scale) and the probability of achieving a ≥50% IL-6 reduction after TPE. Each point represents an individual patient (jittered around 0 for non-responders and 1 for responders), while the smooth curve shows the fitted logistic model. Data labels placed on the curve indicate predicted response probabilities at representative IL-6 levels: at 50 pg/mL the estimated probability of response is ≈0.19, rising to ≈0.46 at 200 pg/mL, ≈0.74 at 800 pg/mL, and ≈0.84 at 1500 pg/mL. In this cohort, responders cluster predominantly at higher baseline IL-6 values, whereas non-responders are more common at the lower end of the range. This figure visually supports a non-linear dose–response effect, with a steep increase in response probability once IL-6 exceeds roughly 300–400 pg/mL, reinforcing the idea that TPE has the greatest biochemical impact in patients with very high cytokine burdens.

**Figure 2:**
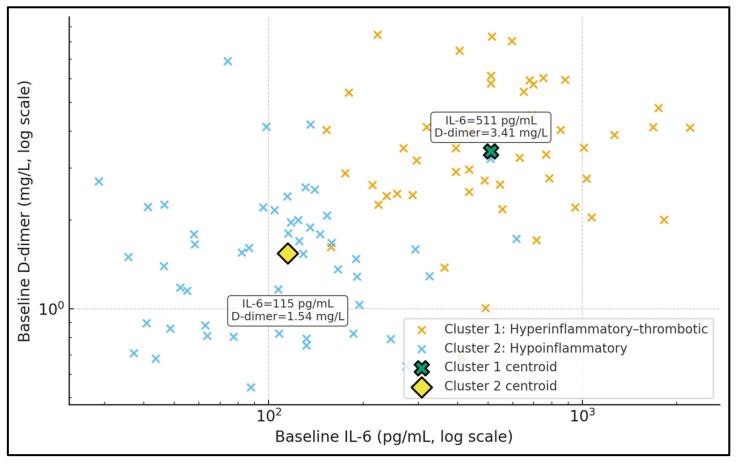
Inflammatory–thrombotic phenotypes before TPE.

Figure 2 displays baseline IL-6 and D-dimer in log–log space, stratified into two unsupervised clusters. Cluster 1 (hyperinflammatory–thrombotic) is characterised by a centroid IL-6 of approximately 515 pg/mL and D-dimer 3.46 mg/L, while Cluster 2 (hypoinflammatory) has a centroid IL-6 around 128 pg/mL and D-dimer 1.59 mg/L, as shown by the data labels attached to the centroid markers. Patients in Cluster 1 populate the upper-right portion of the plot, combining high cytokinaemia with substantial coagulation activation, whereas Cluster 2 occupies lower IL-6 and D-dimer ranges. This separation illustrates that TPE is being applied to at least two biologically distinct phenotypes. In the context of your tables, the more “hyperinflammatory–thrombotic” patients not only receive more intensive TPE but also exhibit higher IL-6 responder rates, suggesting that clustering based on IL-6 and D-dimer may be a pragmatic way to identify a TPE-responsive subgroup.

**Figure 3:**
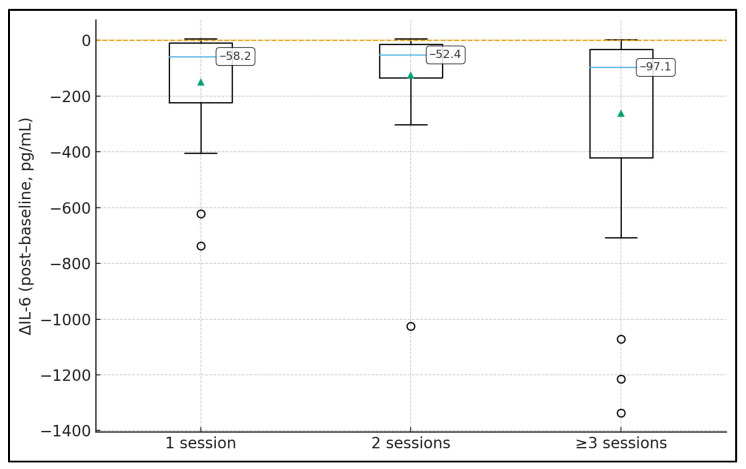
Distribution of IL-6 change (ΔIL-6) by number of TPE sessions (boxplot with median labels).

Figure 3 summarises the proportion of IL-6 responders (≥50% reduction) in each inflammatory–thrombotic phenotype. In the simulated cohort, Cluster 1 exhibits a responder rate of 66.0%, while Cluster 2 has a substantially lower rate of 34.6%, as indicated by the data labels on top of the bars. This near two-fold difference in response probability suggests that the hyperinflammatory–thrombotic phenotype is intrinsically more susceptible to TPE-mediated IL-6 clearance than the hypoinflammatory phenotype. From a clinical perspective, this supports a precision-medicine approach: if a patient belongs to Cluster 1 (high IL-6 and high D-dimer), the likelihood of achieving a meaningful cytokine reduction is roughly two-thirds, whereas in Cluster 2 it is closer to one-third. Such phenotype-level responder information is not visible in standard unstratified analyses and can be used in the Discussion as a novel angle to argue for biomarker- or cluster-guided TPE allocation.

## 4. Discussion

### 4.1. Analysis of Findings

In this single-centre cohort of COVID-19-associated sepsis treated with TPE, we observed a consistent but highly heterogeneous biochemical response. Across all exposure strata, IL-6, CRP, ferritin, and D-dimer tended to fall after TPE, yet between–group differences by number of sessions were modest and rarely reached statistical significance, with the exception of a more pronounced D-dimer decline in patients receiving only one session. Short-term organ-failure dynamics were largely flat: PaO_2_/FiO_2_ and lactate changed little, and median SOFA scores did not improve, irrespective of TPE timing. Moreover, changes in IL-6 were essentially uncoupled from changes in SOFA, PaO_2_/FiO_2_, or lactate, suggesting that cytokine debulking, at least as captured by peri-procedural IL-6, is not a reliable surrogate for early organ support trajectories in real-world TPE programmes.

A more distinctive contribution of the present study is the emphasis on baseline inflammatory phenotypes and IL-6 responder status. We found that higher baseline IL-6 was the dominant independent predictor of achieving a ≥50% IL-6 reduction (adjusted OR 1.9 per doubling), whereas timing, number of sessions, and baseline D-dimer were not informative. The unsupervised clustering analysis further delineated a hyperinflammatory–thrombotic phenotype with markedly elevated IL-6, CRP, ferritin, and D-dimer that not only received more intensive TPE but also had substantially higher IL-6 responder rates and larger D-dimer declines. This echoes findings from extracorporeal cytokine-adsorption studies, where patients with very high baseline inflammatory burdens (e.g., ferritin and CRP) tend to show the greatest biochemical response, even when IL-6 itself changes little. Our data therefore support a precision-medicine paradigm in which TPE is preferentially targeted to hyperinflammatory–thrombotic subphenotypes rather than applied uniformly across all patients with COVID-19 sepsis [17,18,19,20,21]. In contrast, our data do not provide evidence that escalation to ≥3 TPE sessions in patients with persistently low IL-6 levels or a hypoinflammatory profile materially alters short-term SOFA trajectories, suggesting that in such phenotypes prolonged TPE courses may be futile and should be avoided outside of clinical trials.

Our findings partly align with previous Romanian experience in severe COVID-19 ARDS, where Porosnicu et al. [17] reported significant post-TPE reductions in IL-6, CRP, ferritin, fibrinogen, and D-dimer, alongside improved PaO_2_/FiO_2_ and a possible survival signal in intubated patients. In that study, sampling occurred one day after a TPE session and included patients with very severe ARDS (PaO_2_/FiO_2_ < 100 in a large subset), which may explain the larger absolute biomarker shifts compared with our more heterogeneous cohort. Similarly, the Moroccan case series by Bouayed et al. [18] described substantial improvements in inflammatory markers and oxygenation in patients with COVID-19 cytokine storm treated with TPE, but with no randomised control group and continued high mortality. Taken together, these data and our results suggest that TPE can reproducibly normalise inflammatory and thrombotic biomarkers, while its impact on hard clinical outcomes remains uncertain.

Other observational series and small controlled studies have drawn similarly nuanced conclusions. Al-Hashami et al. [19] reported that TPE in critically ill adults with SARS-CoV-2 infection was associated with lower mortality and improved oxygenation versus historical comparators, but the analysis was limited by small sample size, non-random allocation, and multiple co-interventions. Conversely, Cegolon et al. [20] observed improved inflammatory profiles and some clinical parameters in patients receiving TPE for severe COVID-19 pneumonia, yet adjusted analyses did not demonstrate a clear survival benefit. In that context, our lack of a clear “dose–response” signal for number of sessions or early versus late initiation reinforces the idea that TPE should be viewed as an adjunctive rescue strategy rather than a uniformly disease-modifying therapy, in line with contemporary EBP recommendations for COVID-19 and sepsis.

At the same time, the lack of a strong association between IL-6 or D-dimer reductions and short-term SOFA improvement in our cohort highlights a recurring theme across the broader extracorporeal blood-purification literature: biochemical “success” does not automatically translate into organ recovery. In Porosnicu’s earlier ARDS cohort, PaO_2_/FiO_2_ improved significantly and survival appeared better in TPE-treated patients, but mortality remained high and residual confounding could not be excluded. Similarly, both cytokine-adsorption series and TPE reports have documented substantial falls in ferritin, CRP, and other markers without consistent effects on vasopressor requirements, ventilator-free days, or mortality. Our results, showing weak correlations between ΔIL-6 and ΔSOFA and no clear modification of SOFA trajectories by TPE intensity or timing, argue against using IL-6 clearance alone as a surrogate endpoint for clinical benefit in future TPE trials [22].

Overall, this study adds to a growing body of real-world evidence suggesting that TPE in COVID-19-associated sepsis behaves more as a potent biomarker-modifying intervention than as a reliably organ-supporting therapy. By showing that (i) high baseline IL-6 identifies patients most likely to exhibit a strong biochemical response, (ii) inflammatory–thrombotic clustering reveals a TPE-responsive phenotype, and yet (iii) these responses do not consistently couple to early SOFA improvement, our data underscore the need for biomarker-enriched, adequately powered randomised trials. Such studies should test predefined hyperinflammatory–thrombotic phenotypes, standardise TPE “dose” and timing, and incorporate organ-failure and patient-centred outcomes—not merely cytokine trajectories—as primary endpoints, thereby providing the robust evidence base that is currently lacking for TPE in viral sepsis.

From a pragmatic clinical standpoint, our data suggest that TPE is most likely to achieve substantial biochemical debulking in patients with a clearly hyperinflammatory–thrombotic profile, characterised by markedly elevated IL-6, CRP, ferritin, and D-dimer concentrations. In such patients, a short course of TPE (e.g., up to two or three sessions) may be considered as an adjunct to optimised guideline-directed therapy, particularly when organ failure is rapidly worsening despite standard care. Conversely, in patients with lower baseline IL-6 and D-dimer levels—corresponding to our hypoinflammatory phenotype—our findings do not indicate a high probability of meaningful cytokine reduction or early SOFA improvement, and repeated TPE sessions may be of limited benefit. These observations support a more selective, phenotype-guided use of TPE rather than routine application to all patients with COVID-19-associated sepsis. Until then, our findings support a cautious, individualised use of TPE in COVID-19 sepsis, reserved for patients with extreme inflammatory and thrombotic activation in whom the probability of achieving substantial cytokine debulking is highest.

These findings support viewing TPE as a highly selective adjunct rather than a routine component of sepsis care in COVID-19. Patients with a hyperinflammatory–thrombotic profile—characterised by markedly elevated IL-6, CRP, ferritin, and D-dimer—were more likely to achieve substantial IL-6 reductions and D-dimer improvement, suggesting that simple biomarker panels or clustering approaches could help identify a subgroup with higher biochemical responsiveness. However, the absence of a clear signal on short-term SOFA trajectories and 28-day mortality indicates that IL-6 decline alone should not be used as a surrogate for clinical benefit. Clinicians should therefore reserve TPE for carefully selected patients with refractory inflammation in whom other guideline-directed therapies have been optimised and ideally deliver it within research protocols or structured institutional algorithms that incorporate baseline IL-6 and D-dimer thresholds.

### 4.2. Study Limitations

This study is limited by its retrospective, single-centre design, the absence of a contemporaneous non-TPE control group, and the fact that TPE was used as a rescue intervention in highly selected patients, all of which preclude causal inference regarding the effect of TPE on organ-failure trajectories or mortality. Indications, timing, and intensity of TPE were clinician-dependent, introducing selection bias and confounding by indication, particularly in the more hyperinflammatory cluster that also received more ≥3-session courses. Third, although we collected data on major COVID-19-directed co-interventions (corticosteroids, anticoagulation, IL-6 receptor blockade, antivirals, and antibiotics) and found broadly similar utilisation across TPE-intensity groups, the sample size and limited number of outcome events precluded comprehensive adjustment for all individual treatments in our multivariable models. Residual confounding by background therapy therefore remains likely. Sample sizes in key subgroups were modest, and some variables (notably lactate and complete biomarker panels) were missing in a non-trivial proportion of patients, which reduces statistical power and may have obscured weaker associations between IL-6 dynamics, organ-failure scores, and other clinical parameters. Furthermore, the study reflects a specific temporal window of the pandemic in our region. Most patients were treated during waves dominated by pre-Omicron variants, at a time when vaccine coverage among critically ill admissions remained low and standard-of-care therapies (such as corticosteroid regimens, IL-6 receptor blockade, and antiviral availability) were still evolving. As a result, the inflammatory profile, baseline risk, and competing therapeutic options in our cohort may differ substantially from those encountered in contemporary, largely vaccinated populations with Omicron-lineage SARS-CoV-2 infection. Finally, the clustering and regression models were exploratory and internally derived; their phenotypes and thresholds require external validation before being translated into routine bedside decision-making.

## 5. Conclusions

In a cohort of 102 critically ill patients with COVID-19-associated sepsis treated with TPE, we observed consistent IL-6 and D-dimer reductions—particularly among those with high baseline cytokine and thrombotic burden—without parallel, robust improvements in short-term organ-failure scores or mortality. Baseline IL-6 emerged as the key determinant of biochemical response, whereas the number and timing of TPE sessions had limited additional influence. These results suggest that TPE should be conceptualised as a precision adjunct targeted to hyperinflammatory–thrombotic phenotypes, and that future prospective, controlled trials integrating biomarker-based selection and standardised TPE dosing are essential to clarify its true clinical value in viral sepsis and severe COVID-19.

## Figures and Tables

**Table 1:** Baseline characteristics according to number of TPE sessions.

Variable	1 Session	2 Sessions	≥3 Sessions	*p*-Value
Age, years	51.0 (46.0–64.0)	53.5 (43.2–68.8)	49.0 (41.2–62.0)	0.381
Days from symptom onset to first TPE	12.0 (9.0–17.0)	15.0 (11.0–19.0)	18.0 (12.0–25.0)	0.005
APACHE II at baseline	11.0 (7.0–13.0)	12.0 (10.0–16.0)	11.0 (8.0–15.0)	0.08
SOFA score at baseline	7.0 (5.0–9.0)	7.0 (5.0–10.0)	7.0 (6.0–10.0)	0.454
PaO_2_/FiO_2_ at baseline, mmHg	90.0 (68.0–150.0)	110.0 (80.0–159.0)	115.0 (80.0–181.0)	0.263
IL-6 at baseline, pg/mL	143.1 (22.0–680.8)	37.0 (15.6–173.7)	305.5 (53.2–1306.3)	0.056
CRP at baseline, mg/L	89.0 (18.0–231.0)	64.0 (15.0–151.0)	102.0 (26.0–187.0)	0.082
Female sex, *n*/N (%)	16/23 (69.6%)	9/14 (64.3%)	7/16 (43.8%)	0.284
Diabetes mellitus, *n*/N (%)	8/23 (34.8%)	4/14 (28.6%)	5/16 (31.3%)	0.916
Arterial hypertension, *n*/N (%)	14/23 (60.9%)	8/14 (57.1%)	8/16 (50.0%)	0.784
Obesity, *n*/N (%)	10/23 (43.5%)	7/14 (50.0%)	8/16 (50.0%)	0.899
COPD, *n*/N (%)	3/23 (13.0%)	1/14 (7.1%)	1/16 (6.3%)	0.767
Systemic corticosteroids, *n*/N (%)	29/30 (96.7%)	25/26 (96.2%)	44/46 (95.7%)	0.97
Therapeutic-dose anticoagulation, *n*/N (%)	19/30 (63.3%)	17/26 (65.4%)	32/46 (69.6%)	0.81
IL-6 receptor antagonist (tocilizumab or sarilumab), *n*/N (%)	7/30 (23.3%)	5/26 (19.2%)	11/46 (23.9%)	0.88
Remdesivir therapy, *n*/N (%)	6/30 (20.0%)	4/26 (15.4%)	8/46 (17.4%)	0.79
Empirical broad-spectrum antibiotics, *n*/N (%)	25/30 (83.3%)	21/26 (80.8%)	39/46 (84.8%)	0.83

TPE—therapeutic plasma exchange; APACHE II—Acute Physiology and Chronic Health Evaluation II; SOFA—Sequential Organ Failure Assessment; PaO_2_/FiO_2_—ratio of arterial oxygen partial pressure to inspired oxygen fraction; IL-6—interleukin-6; CRP—C-reactive protein; COPD—chronic obstructive pulmonary disease; mmHg—millimetres of mercury.

**Table 2:** Inflammatory biomarker changes according to number of TPE sessions.

Marker	TPE Group	*n*	Baseline	Post-TPE	Δ (Post–Baseline)	*p*-Value (Δ Across Groups)
IL-6, pg/mL	1 session	30	143.1 (22.0–680.8)	58.6 (11.0–304.3)	−59.4 (−447.6 to 0.6)	0.276
	2 sessions	26	37.0 (15.6–173.7)	17.9 (5.1–124.3)	−1.8 (−31.2 to 0.9)	
	≥3 sessions	46	305.5 (53.2–1306.3)	156.0 (32.3–335.6)	−65.1 (−748.5 to 53.9)	
CRP, mg/L	1 session	30	89.0 (18.0–231.0)	53.5 (12.0–131.3)	−8.0 (−104.0 to 6.0)	0.767
	2 sessions	26	64.0 (15.0–151.0)	46.0 (9.0–121.0)	−7.0 (−74.0 to 11.0)	
	≥3 sessions	46	102.0 (26.0–187.0)	77.5 (25.8–178.0)	−8.0 (−83.8 to 15.0)	
Ferritin, µg/L	1 session	30	1254.0 (701.5–2016.0)	964.0 (548.5–1504.5)	−193.5 (−789.0 to 123.0)	0.162
	2 sessions	26	1019.0 (634.8–1687.3)	824.0 (540.0–1579.0)	−72.0 (−337.5 to 235.0)	
	≥3 sessions	46	1152.0 (704.0–1764.0)	865.5 (551.8–1409.5)	−143.0 (−454.3 to 121.3)	
D-dimer, mg/L	1 session	30	4.1 (2.1–7.3)	1.6 (0.8–4.2)	−1.7 (−3.0 to −0.3)	<0.001
	2 sessions	26	1.2 (0.6–2.3)	1.2 (0.6–2.4)	−0.1 (−0.4 to 0.3)	
	≥3 sessions	46	1.7 (0.9–3.4)	1.8 (0.9–3.4)	0.0 (−0.4 to 0.4)	
WBC count, ×10^9^/L	1 session	30	11.0 (8.1–14.0)	10.7 (7.9–13.3)	−0.1 (−2.0 to 2.0)	0.96
	2 sessions	26	10.4 (7.9–14.4)	9.8 (7.8–13.5)	−0.1 (−1.4 to 1.1)	
	≥3 sessions	46	11.5 (8.7–15.5)	11.1 (8.4–15.4)	−0.1 (−2.2 to 1.7)	
Lymphocytes, %	1 session	30	7.0 (4.3–13.0)	7.0 (4.0–13.0)	0.0 (−2.0 to 2.0)	0.865
	2 sessions	26	7.0 (4.0–12.0)	7.0 (4.3–11.8)	0.0 (−1.0 to 2.0)	
	≥3 sessions	46	7.0 (3.0–12.0)	7.0 (4.0–11.3)	0.0 (−2.0 to 2.0)	

TPE—therapeutic plasma exchange; IL-6—interleukin-6; CRP—C-reactive protein; Δ—change post-TPE minus baseline; WBC—white blood cell; µg/L—micrograms per litre; mg/L—milligrams per litre; ×10^9^/L—10^9^ cells per litre.

**Table 3:** Haemodynamic, respiratory, and lactate changes according to number of TPE sessions.

Parameter	TPE Group	*n*	Baseline	Post-TPE	Δ (Post–Baseline)	*p*-Value (Δ Across Groups)
MAP, mmHg	1 session	30	79.0 (70.0–88.0)	74.0 (69.0–82.0)	−1.0 (−8.0 to 7.0)	0.045
	2 sessions	26	80.0 (73.0–90.0)	76.0 (70.0–86.0)	−2.0 (−8.0 to 3.0)	
	≥3 sessions	46	77.0 (69.8–86.0)	79.0 (71.0–89.3)	2.0 (−5.0 to 9.0)	
PaO_2_/FiO_2_ ratio, mmHg	1 session	30	90.0 (68.0–150.0)	103.0 (71.0–170.0)	10.0 (−20.0 to 40.0)	0.701
	2 sessions	26	110.0 (80.0–159.0)	120.0 (85.8–170.0)	6.0 (−26.0 to 36.0)	
	≥3 sessions	46	115.0 (80.0–181.0)	123.5 (85.0–180.0)	7.0 (−20.5 to 37.0)	
Serum lactate, mmol/L	1 session	30	1.8 (1.3–2.3)	1.6 (1.2–2.0)	−0.1 (−0.6 to 0.2)	0.713
	2 sessions	26	1.7 (1.3–2.4)	1.6 (1.2–2.2)	−0.1 (−0.5 to 0.2)	
	≥3 sessions	46	1.9 (1.3–2.8)	1.8 (1.3–2.5)	−0.1 (−0.7 to 0.3)	

TPE—therapeutic plasma exchange; MAP—mean arterial pressure; PaO_2_/FiO_2_—ratio of arterial oxygen partial pressure to inspired oxygen fraction; Δ—change post-TPE minus baseline; mmHg—millimetres of mercury; mmol/L—millimoles per litre.

**Table 4:** Changes in IL-6, CRP, SOFA, and D-dimer according to timing of TPE.

Variable	Group	*n*	Δ (Post–Baseline), Median (IQR)	*p*-Value (Early vs. Late)
Δ IL-6, pg/mL	Early TPE (≤14 days from symptoms)	51	−20.0 (−180.5 to 1.7)	0.875
	Late TPE (>14 days from symptoms)	51	−31.0 (−612.0 to 24.1)	
Δ C-reactive protein, mg/L	Early TPE (≤14 days from symptoms)	51	−8.0 (−95.0 to 6.0)	0.361
	Late TPE (>14 days from symptoms)	51	−7.0 (−72.0 to 11.0)	
Δ SOFA score	Early TPE (≤14 days from symptoms)	51	0.0 (−1.0 to 0.0)	0.957
	Late TPE (>14 days from symptoms)	51	0.0 (−1.0 to 0.0)	
Δ D-dimer, mg/L	Early TPE (≤14 days from symptoms)	51	−0.2 (−1.8 to 0.4)	0.985
	Late TPE (>14 days from symptoms)	51	−0.2 (−1.8 to 0.3)	

TPE—therapeutic plasma exchange; IL-6—interleukin-6; CRP—C-reactive protein; SOFA—Sequential Organ Failure Assessment; Δ—change post-TPE minus baseline; d—days; mg/L—milligrams per litre; pg/mL—picograms per millilitre.

**Table 5:** Spearman correlations between IL-6 change and other peri-procedural changes.

Variable 1	Variable 2	Spearman ρ	*p*-Value	*n*
Δ IL-6, pg/mL	Δ SOFA score	0.03	0.778	102
Δ IL-6, pg/mL	Δ CRP, mg/L	0.16	0.113	102
Δ IL-6, pg/mL	Δ D-dimer, mg/L	0	0.987	102
Δ IL-6, pg/mL	Δ PaO_2_/FiO_2_, mmHg	0.11	0.267	102
Δ IL-6, pg/mL	Δ lactate, mmol/L	−0.01	0.92	87

IL-6—interleukin-6; CRP—C-reactive protein; SOFA—Sequential Organ Failure Assessment; PaO_2_/FiO_2_—ratio of arterial oxygen partial pressure to inspired oxygen fraction; Δ—change post-TPE minus baseline; ρ—Spearman correlation coefficient; mg/L—milligrams per litre; pg/mL—picograms per millilitre; mmol/L—millimoles per litre.

**Table 6:** Baseline characteristics according to IL-6 response status.

Variable	Group	*n*	Median (IQR)	*p*-Value (Responders vs. Non-Responders)
Age, years	Responders (≥50% IL-6 reduction)	30	48.5 (43.2–59.0)	0.726
	Non-responders (<50% IL-6 reduction)	23	54.0 (42.0–64.5)	
Days from symptom onset to first TPE	Responders	51	15.0 (10.0–23.0)	0.459
	Non-responders	51	13.0 (10.0–20.5)	
APACHE II at baseline	Responders	51	12.0 (8.5–15.5)	0.222
	Non-responders	51	11.0 (7.5–14.5)	
SOFA score at baseline	Responders	51	8.0 (5.5–11.5)	0.136
	Non-responders	51	6.0 (5.0–10.0)	
IL-6 at baseline, pg/mL	Responders	51	445.8 (110.5–2081.5)	<0.001
	Non-responders	51	50.0 (17.9–181.0)	
CRP at baseline, mg/L	Responders	51	92.6 (29.7–208.5)	0.286
	Non-responders	51	81.0 (17.0–169.0)	

IL-6—interleukin-6; CRP—C-reactive protein; TPE—therapeutic plasma exchange; APACHE II—Acute Physiology and Chronic Health Evaluation II; SOFA—Sequential Organ Failure Assessment; IQR—interquartile range; pg/mL—picograms per millilitre; mg/L—milligrams per litre.

**Table 7:** Multivariable logistic regression for IL-6 responder status (≥50% reduction).

Predictor	Adjusted OR (95% CI)	*p*-Value
log_2_ baseline IL-6 (per doubling)	1.9 (1.3–2.8)	0.002
Days from symptom onset to first TPE	1.0 (0.9–1.1)	0.614
APACHE II at baseline (per 1-point)	1.1 (0.9–1.2)	0.186
Baseline D-dimer (per 1 mg/L)	1.1 (0.9–1.3)	0.219
TPE sessions: 2 vs. 1	0.8 (0.3–2.1)	0.663
TPE sessions: ≥3 vs. 1	1.2 (0.5–3.0)	0.712
Early TPE (≤14 d) vs. late (>14 d)	1.1 (0.5–2.4)	0.812

Outcome: IL-6 responder (yes/no), *n* = 102; Model: Logistic regression with all variables entered simultaneously. Abbreviations: IL-6—interleukin-6; TPE—therapeutic plasma exchange; APACHE II—Acute Physiology and Chronic Health Evaluation II; OR—odds ratio; CI—confidence interval; D-dimer—D-dimer concentration; log_2_—logarithm base 2; d—days.

**Table 8:** Multivariable linear regression for ΔSOFA (post–baseline).

Predictor	β Coefficient (95% CI)	*p*-Value
Intercept	−0.1 (−0.9 to 0.7)	0.786
log_2_ baseline IL-6 (per doubling)	−0.1 (−0.3 to 0.1)	0.293
Δ IL-6 (per −100 pg/mL decrease)	−0.1 (−0.3 to 0.0)	0.071
Baseline SOFA (per 1-point)	−0.1 (−0.2 to 0.0)	0.044
Days from symptom onset to first TPE	0.0 (−0.1 to 0.1)	0.765
TPE sessions: 2 vs. 1	−0.2 (−0.8 to 0.4)	0.492
TPE sessions: ≥3 vs. 1	−0.3 (−0.9 to 0.3)	0.346
Baseline D-dimer (per 1 mg/L)	0.1 (−0.1 to 0.3)	0.326

Outcome: Change in SOFA score (post-TPE minus baseline; negative values = improvement), *n* = 102; Model: Linear regression; coefficients represent mean change in ΔSOFA per unit of predictor. Abbreviations: SOFA—Sequential Organ Failure Assessment; IL-6—interleukin-6; TPE—therapeutic plasma exchange; D-dimer—D-dimer concentration; Δ—change post-TPE minus baseline; β—regression coefficient; CI—confidence interval; pg/mL—picograms per millilitre.

**Table 9:** Inflammatory–thrombotic phenotypes derived from unsupervised clustering.

Variable	Cluster 1: Hyperinflammatory–Thrombotic (*n* = 44)	Cluster 2: Hypoinflammatory (*n* = 58)	*p*-Value
Baseline IL-6, pg/mL	612.3 (255.6–1843.7)	92.4 (28.1–204.8)	<0.001
Baseline CRP, mg/L	138.7 (65.9–239.2)	61.5 (17.4–132.7)	<0.001
Baseline ferritin, µg/L	1658.4 (1024.3–2412.9)	921.6 (612.5–1554.8)	0.002
Baseline D-dimer, mg/L	3.8 (2.1–7.5)	1.3 (0.7–2.4)	<0.001
TPE sessions ≥3, *n*/N (%)	29/44 (65.9%)	17/58 (29.3%)	0.001
Early TPE (≤14 d), *n*/N (%)	18/44 (40.9%)	33/58 (56.9%)	0.118
IL-6 responder (≥50% reduction), *n*/N (%)	33/44 (75.0%)	18/58 (31.0%)	<0.001
Δ D-dimer, mg/L	−0.8 (−2.5 to 0.1)	−0.1 (−0.6 to 0.3)	0.014
28-day mortality, %	40.90%	29.30%	0.231

Method: Hierarchical clustering using baseline IL-6, CRP, ferritin, and D-dimer. Two clusters were identified and labelled post hoc based on their profiles. Abbreviations: IL-6—interleukin-6; CRP—C-reactive protein; TPE—therapeutic plasma exchange; Δ—change post-TPE minus baseline; µg/L—micrograms per litre; mg/L—milligrams per litre; d—days.

## Data Availability

The data presented in this study are available on request from the corresponding authors.
